# An Interpenetrated Anionic MOF Featuring Amide-Functionalized Pores for CO_2_ and Methylene Blue Adsorption

**DOI:** 10.1021/acs.inorgchem.5c02360

**Published:** 2025-06-24

**Authors:** Tuğba Alp Arici, Melike Şevik, Enes Kavak, Mürsel Arici

**Affiliations:** a Department of Chemical Technology, Emet Vocational School, 52956 Kütahya Dumlupınar University, Kütahya 43700, Türkiye; b Department of Chemistry, Faculty of Science, 53004 Eskişehir Osmangazi University, Eskişehir 26040, Türkiye

## Abstract

A multifunctional metal–organic framework (MOF), {(pbisoixH_2_)­[Zn­(μ_4_-BDPFA)]·2DMF·4H_2_O}_*n*_ (ESOGU-3) (BDPFAH_4_: *N* ^1^,*N* ^4^-bis­(3,5-dicarboxyphenyl)­fumaramide, pbisoixH_2_: protonated form of 1,4-bis­(2-isopropylimidazol-1-ylmethyl)­benzene) was synthesized and thoroughly characterized using a variety of analytical techniques. X-ray crystallographic analysis revealed that the anionic 3D framework is constructed from Zn­(II) ions coordinated with BDPFA^4–^ ligands, with the framework’s negative charge balanced by protonated pbisoixH_2_ counterions. The anionic frameworks interpenetrated each other to form a 2-fold interpenetrating porous 3D framework. Thanks to its porous architecture and stability in water, ESOGU-3 was evaluated for gas and dye adsorption performance. The single-component gas adsorption results showed that ESOGU-3 with a BET surface area of 805.73 m^2^/g displayed selective CO_2_ adsorption (3.82 mmol/g at 100 kPa) over CH_4_ and N_2_ at 273 K. The enhanced CO_2_ adsorption capacity was attributed to amide-functionalized groups within the framework. Furthermore, ESOGU-3, featuring an anionic framework, exhibited selective adsorption toward the smaller cationic methylene blue (MB), achieving a maximum adsorption capacity of 415.78 mg/g. This selectivity is attributed to favorable electrostatic interactions with MB over anionic dyes and size-exclusion effects that limit the adsorption of the larger rhodamine B (RhB) dye.

## Graphical abstract

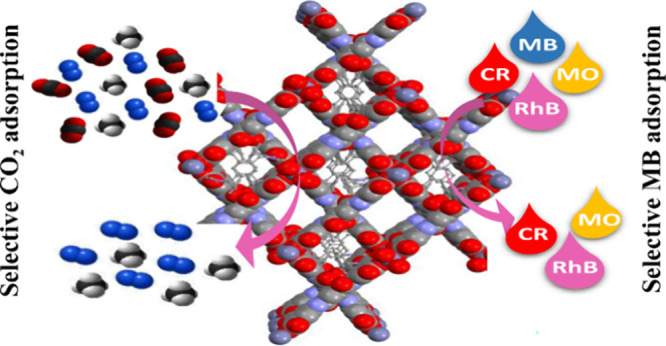

## 1 Introduction

MOFs, inorganic–organic crystalline materials, have attracted significant attention due to their high surface areas, tunable pore sizes, and permanent porosity.^1–3^ These properties make MOFs highly versatile for diverse applications, including adsorption/separation, catalysis, and sensing.^3–6^ Especially, MOFs have been extensively investigated as solid adsorbents for capturing CO_2_, one of the primary greenhouse gases contributing to global warming, because of their high capacity and selectivity compared to other porous materials.^7–9^ Several parameters influence the adsorption performance of the CO_2_ of MOFs. According to the literature, enhancing CO_2_ uptake often involves functionalizing MOF surfaces with polar groups such as amines (−NH_2_), hydroxyl (−OH), and amides (−NH–CO−). MOFs containing amide groups, particularly, have been widely developed due to their strong affinity for CO_2_ through Lewis acid–base interactions.^10–15^ Among these, NJU-BAI and HNUST-MOFs have shown promising performance in both CO_2_ adsorption and separation.^13,14,16–18^ Additionally, open metal sites (OMSs) in MOFs play a crucial role in enhancing CO_2_ uptake, primarily due to strong interactions between the metal centers and CO_2_ molecules.^17,19^ MOFs based on copper­(II) and magnesium­(II) with accessible OMSs have demonstrated notably high adsorption capacities.^20,21^ However, many MOFs with OMSs suffer from instability under humidity conditions. To address this limitation, structural stability can be enhanced through strategies such as incorporating hydrophobic functional groups, formation of interpenetration in the final structure, or employing mixed-ligand approaches.^22–24^ In mixed-ligand coordination polymers, porosity is often reduced due to the incorporation of a secondary linker. As secondary linkers, bis­(pyridine) and bis­(imidazole) derivative ligands have been widely preferred in mixed-ligand compounds. Bis­(imidazole) derivatives typically lead to nonporous structures, whereas bis­(pyridine) derivatives have a higher potential to form porous frameworks.^9,25^ For example, TMU frameworks including bis­(pyridine) derivatives displayed permanent porosity and demonstrated efficient CO_2_ adsorption.^26^ In the mixed-ligand compounds, bis­(imidazole) derivative linkers rarely result in a porous structure. However, in our previous study on mixed-ligand compounds, bis­(imidazole) linker was protonated in the reaction medium and acted as a counterion in the presence of an amide-functionalized tetracarboxylic acid and Zn­(II). This resulted in a porous structure, and the compound exhibited efficient CO_2_ adsorption capacity.^23^

Dyes, widely used in the textile industry, are major contributors to environmental pollution due to their toxicity, carcinogenicity, and high stability in aqueous environments.^27^ Consequently, the effective removal of dyes from water is of critical importance. MOFs have demonstrated excellent performance in this area, offering high dye removal efficiency.^28^ Beyond their efficiency, MOFs also enable selective adsorption based on dye size or charge, owing to their tunable surface properties.^29^ Our group has previously reported several anionic MOFs with charged surfaces capable of selectively adsorbing cationic dyes based on size exclusion and electrostatic interactions.^23,30,31^

Taking the above into account, in this work, a mixed-ligand anionic MOF, {(pbisoixH_2_)­[Zn­(μ_4_-BDPFA)]·2DMF·4H_2_O}_*n*_ (ESOGU-3, where ESOGU refers to Eskişehir Osmangazi University), was prepared using pbisoix and amide-decorated tetracarboxylic acid in the presence of Zn­(II), similar to our previous study.^23^ The *N* ^1^,*N* ^4^-bis­(3,5-dicarboxyphenyl)­fumaramide (BDPFAH_4_) ligand can coordinate to metal ions through eight oxygen atoms from its four carboxylate groups. Additionally, the semiflexible pbisoix linker acted as a counterion, as predicted (Scheme 1). The multifunctional ESOGU-3 was employed for the adsorption of CO_2_, CH_4_, and N_4_ gases as well as for the adsorption of dyes with varying charges and molecular sizes. The interpenetrated ESOGU-3 exhibited high CO_2_ selectivity over other gases via Lewis acid–base interactions and showed selective adsorption toward small cationic MB over larger cationic RhB and anionic dyes (methyl orange (MO) and congo red (CR)), primarily due to electrostatic interactions and size selectivity.

**Scheme 1:**
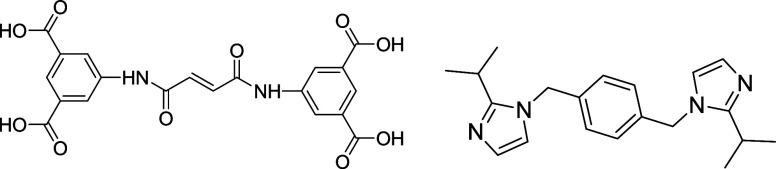
Structures of BDPFAH_4_ and Pbisoix Linkers

## 2 Experimental Section

### 2.1 Materials and Physical Measurements

The chemicals used for the synthesis were obtained from Sigma-Aldrich. The linkers were prepared based on established literature methods.^30,32,33^ Information on physical measurements and the experimental procedures for adsorption studies is provided in the Supporting Information (SI) file.

### 2.2 ESOGU-3’s Synthesis

BDPFAH_4_ (0.044 g, 0.1 mmol), pbisoix (0.032 g, 0.1 mmol), and ZnCl_2_ (0.028 g, 0.2 mmol) were stirred in the mixture of DMF/H_2_O (9:3, v:v, mL) at room temperature for 30 min, then 60 μL of HNO_3_ (6.0 M) was added to the previous mixture to obtain a clear solution. The resulting solution was placed in the closed-capped bottle (20 mL) and heated at 80 °C for 3 days, yielding yellow crystals. Yield: 79% (based on BDPFAH_4_). Anal. Calcd for C_46_H_60_N_8_O_16_Zn: C, 52.80; H, 5.78; N, 10.71%. Found: C, 52.16; H, 5.49; N, 10.89%. FT-IR (KBr, cm^–1^): 3454 m, 3260 m, 3151 m, 3132s, 3041 m, 2976 m, 2937 m, 2877 m, 1668 vs, 1632 vs, 1582 vs, 1404 vs, 1336 s, 1175 m, 1103 m, 963 m, 912 m, 781 s, cm^–1^

## 3 Results and Discussion

### 3.1 Synthesis and Characterization

ESOGU-3 with an anionic skeleton was prepared with an amide containing tetracarboxylic acid and pbisoix ligands using zinc salt as a single crystal. The compound was characterized using several techniques. Elemental analysis results agree with the proposed formula. In the FT-IR spectrum, the broad peak observed at 3454 cm^–1^ corresponds to the ν­(O–H) stretching vibrations of the water molecules. The weak peaks appearing between 3151 and 2877 cm^–1^ are related to aromatic and aliphatic ν­(C–H) stretching vibrations. In the compound, the asymmetric and symmetric ν­(COO^–^) vibrations of the deprotonated carboxylate groups of the BDPFA^4–^ linker are observed at 1582 and 1336 cm^–1^, respectively. Moreover, the carbonyl stretching vibrations of DMF molecule or amide group of BDPFA^4–^ appear at 1668 cm^–1^ (Figure S1).

The structure of ESOGU-3 was similar to the reported compound, ESOGU-2.^23^ Figure 1 shows the crystal structure, highlighting the coordination environment around the Zn1 center along with the atomic numbering, and Table 1 lists the crystal data together with the structure refinement parameters. The selected bond distances, angles, and hydrogen-bond geometry for ESOGU-3 are presented in Table S1. ESOGU-3 crystallizes in an orthorhombic system with the space group of *Ccce*. There are half zinc ion, half BDPFA^4–^ and half protonated pbisoixH_2_ ligands and solvent molecules in the ESOGU-3’s asymmetric unit. The solvent molecules are removed from the structure using the solvent-masked protocol in Olex2.^34^ The quantity of solvent molecules is then determined through TG and elemental analyses. Based on elemental analysis and TG results and solvent mask calculations (which detected 1008 electrons in one void per unit cell), we confirmed that two DMF and four water molecules are present. Zinc ions coordinate to four carboxylate oxygen atoms from four different BDPFA^4–^ ligands to form a distorted tetrahedral geometry (Figure 1).

**Figure 1:**
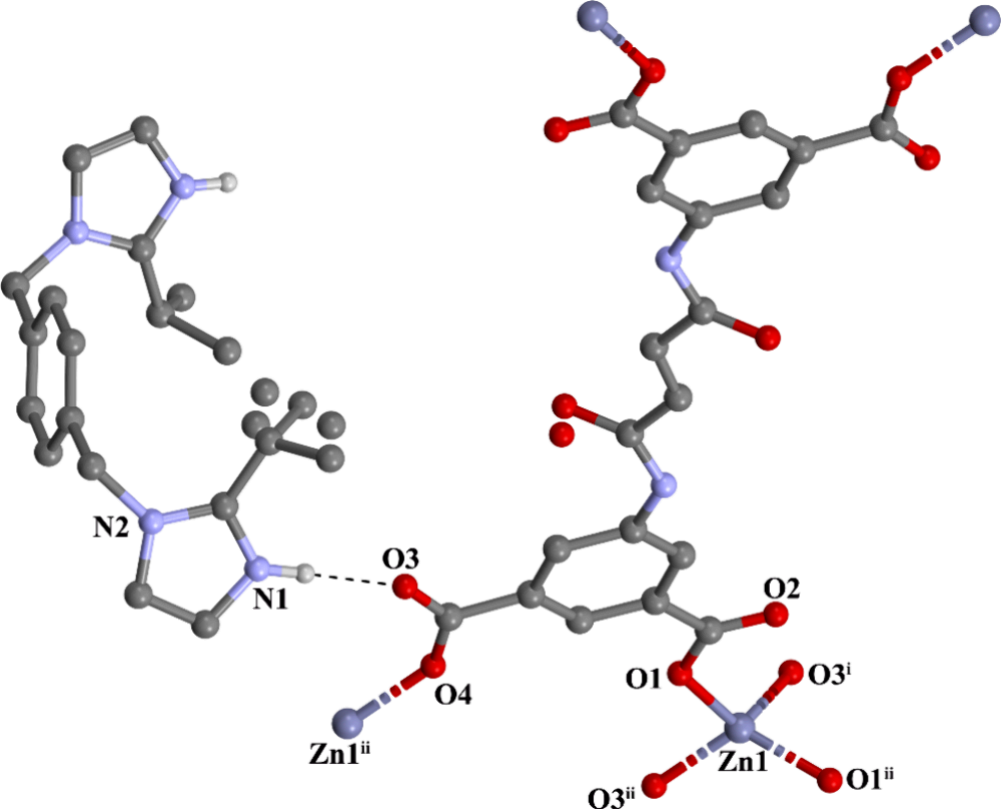
Coordination environment around Zn1 with the atom numbering (hydrogen atoms were omitted for clarity) ((i) *x*, −*y* + 1, *z*–1/2; (ii) −*x* + 1, −*y* + 1, −*z* + 1; (iii) −*x* + 1, *y*, −*z* + 1/2).

**Table 1:** Crystal Data and Structure Refinement Parameters for ESOGU-3

	ESOGU-3
CCDC	2453346
empirical formula	C_40_H_38_N_6_O_10_Zn
formula weight	1046.39
crystal system	orthorhombic
space group	*Ccce*
*a (*Å)	24.6085 (6)
*b (*Å)	29.9779 (7)
*c (*Å)	14.8573 (3)
α*(*°)	90
β *(*°)	90
γ*(*°)	90
*V (*Å^3^)	10960.4 (4)
*Z*	8
*D*_c_ (g cm^–3^)	1.268
μ (mm^–1^)	1.22
θ range (°)	3.8–77.9
measured refls.	148637
independent refls.	5845
*R*_int_	0.098
S	1.07
R1/wR2	0.050/0.144
Δρ_max_/Δρ_min_ (eÅ^–3^)	0.34/ −0.46

The BDPFA^4–^ ligands connect to four zinc ions as a monodentate to generate an anionic 3D framework that includes rectangular channels with dimensions of 7.445···19.392 Å^2^ (Zn···Zn distances) (along with the bc-plane) and square channels with dimensions of 19.392···19.392 Å^2^ (Zn···Zn distances) (along with the *c*-axis) (Figure 2). The charge in the anionic framework is kept balanced because the pbisoix ligand gets protonated.

**Figure 2:**
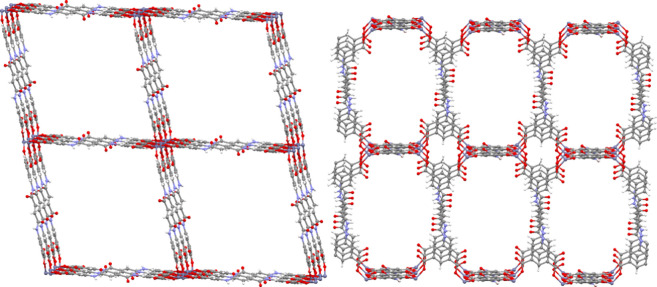
3D anionic porous framework with square and rectangular channels.

Due to the presence of large channels, the structure forms a 2-fold interpenetrated 3D network, where identical units interlock together (Figure 3a). Hydrogen bonding from the protonated pbisoixH_2_ ligands helps stabilize this interpenetrating framework. Even with this interpenetration, the structure still features square-shaped channels filled with solvent molecules and protonated ligands (Figure 3a). Following solvent removal, PLATON analysis indicates that the total solvent-accessible volume is 3750.2 Å^3^, accounting for 34.2% of the unit cell volume. ESOGU-3 adopts a pts topology with a point symbol of 4^2^.8^4^, just like the one seen in ESOGU-2 (Figure 3b).

**Figure 3:**
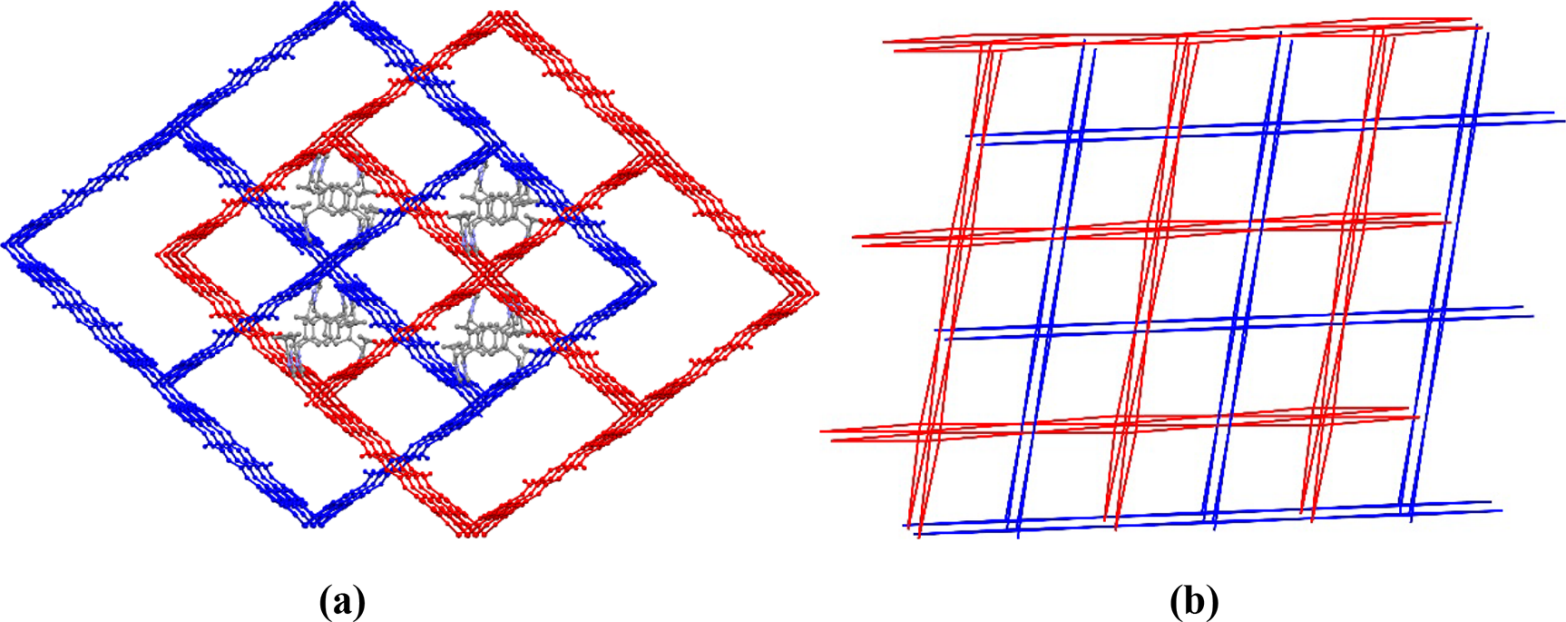
2-fold interpenetrating 3D + 3D→3D framework (**a**) and a topologically simplified representation (**b**) of ESOGU-3.

PXRD patterns of ESOGU-3 were recorded to control the phase purity of the bulk compounds (Figure 4). The simulated PXRD pattern, generated using single-crystal X-ray data in the Mercury program, lined up well with the experimental results, showing that the bulk compound is phase pure. Moreover, to determine the stability of the framework, PXRD patterns of activated ESOGU-3, which was immersed in methanol and then heated at 90 °C, and ESOGU-3@water immersed in water for 2 days were recorded. PXRD patterns of them were similar to that of the as-synthesized ESOGU-3, indicating the stability in water and after the activation process (Figure 4). In the literature, anionic MOFs typically contain dimethylamine cations or protonated N-donor linkers.^23,35–37^ In particular, anionic MOFs constructed with Zn­(II) or Cd­(II) ions often exhibit water stability, as these cations complete their coordination spheres within the framework. Additionally, the stability of anionic MOFs can be attributed to the formation of the framework from poly­(carboxylic acid)­s and metal ions, which reinforces the structural integrity.

**Figure 4:**
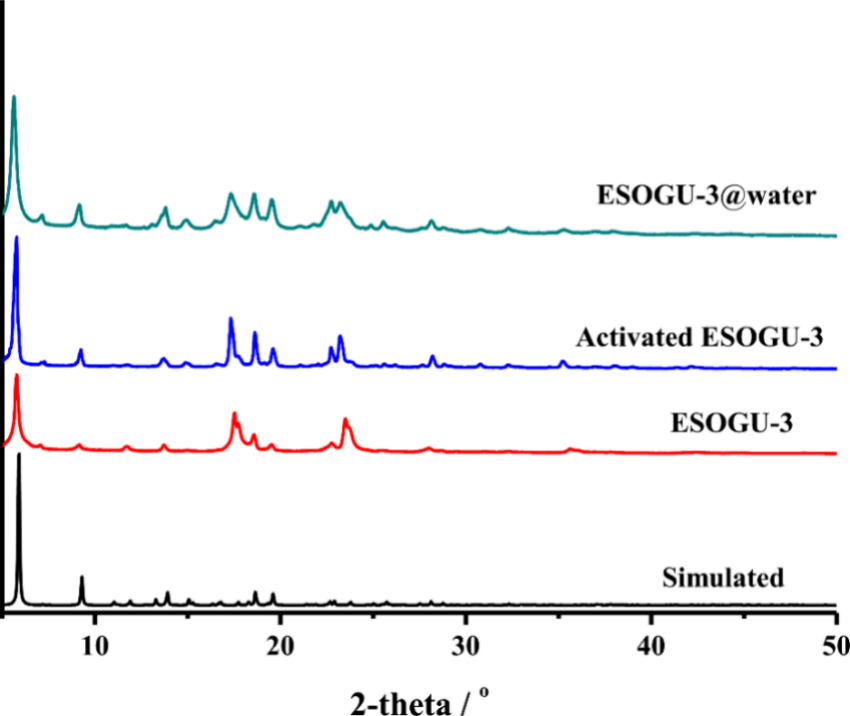
PXRD patterns of simulated, pristine, and activated ESOGU-3 and ESOGU-3 exposed to water.

Thermogravimetric analysis of ESOGU-3 was carried out between 30 and 700 °C to evaluate its thermal stability and determine the solvent removal temperature (Figure S2). The TG curve reveals an initial weight loss of 21.25%, attributed to the release of two DMF and four water molecules (calculated: 20.86%). Following the release of these solvent molecules, the framework remains thermally stable up to 275 °C. Further heating leads to decomposition, resulting in the formation of ZnO as the final residue (observed: 9.34%; calculated: 7.74%).

### 3.2 Gas Adsorption Results

According to the SCXRD result, ESOGU-3 seems to have potential pores for the gas uptake despite the interpenetration. Hence, N_2_ adsorption/desorption isotherms of ESOGU-3 were obtained to determine the permanent porosity at 77 K and 0–1 bar. As is known, the pores of porous compounds are usually filled with solvent molecules. Based on the crystal structure, the pores of ESOGU-3 are occupied by protonated pbisoix ligands and solvent molecules. During the activation process, only the solvent molecules are removed. As a result, half of the pores in ESOGU-3 remain filled with protonated pbisoix ligands, while the other half are accessible and available for gas adsorption. After the removal of solvent molecules, ESOGU-3 uptakes 241 cm^3^/g N_2_ at 1.0 bar and 77 K and the N_2_ sorption isotherm exhibits a reversible type-I isotherm with little hysteresis reflecting the nature of the micropores with permanent microporosity (Figure 5). The complete activation of ESOGU-3 is confirmed by TG analysis (Figure S2). BET and Langmuir surface areas calculated from the isotherm are 805.73 and 1019 m^2^/g, respectively. The surface area of ESOGU-3 is lower than that of ESOGU-2 (1112.77 m^2^/g). The reason for the low surface area of ESOGU-3 can be assigned to the skeletal structure of the BDPFA linker. In ESOGU-2 and ESOGU-3, the linkers contain thiophene rings and but-2-ene groups on their backbones, respectively. As is known, pore walls have an important effect on the gas adsorption. In the ESOGU-2, aromatic thiophene rings on the pore walls enhance the N_2_ adsorption when compared to but-2-ene, which is an acyclic alkene on the pore walls of ESOGU-3. However, the surface area of the compound is still comparable with the reported interpenetrated compounds.^22,23,38,39^ The pore-size distribution of ESOGU-3, based on N_2_ adsorption data and analyzed using the DFT model in Micromeritics Tristar II (assuming a slit-pore geometry), indicates a narrow pore width range of approximately 10.0 Å, confirming the microporosity of the compound (Figure 5, inset).

**Figure 5:**
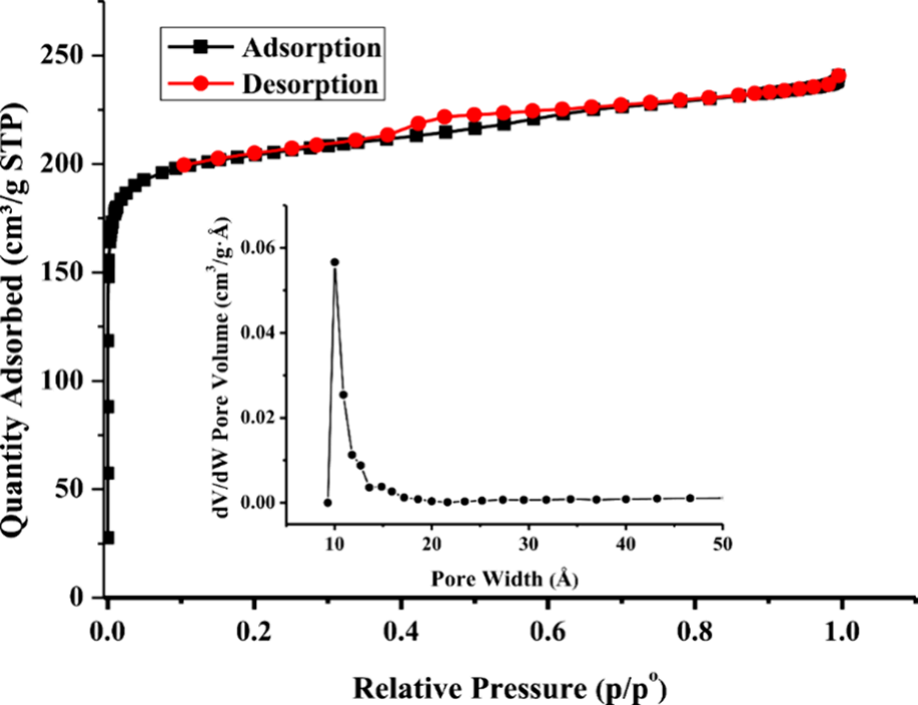
N_2_ adsorption–desorption isotherms at 77 K for ESOGU-3 (inset: pore-size distributions)

As known, the MOFs bearing amide (−CONH−) groups have been extensively synthesized to enhance CO_2_ adsorption due to their interactions with CO_2_ via dipole–quadrupole interactions and the high CO_2_ adsorption capacities with MOFs were reported thanks to amide groups.^18,40^ Hence, single-component CO_2_, CH_4_, and N_2_ adsorption/desorption isotherms are collected at 273 K until 120 kPa (Figure 6). As seen in Figure 6, the adsorption amounts of CO_2_, CH_4_, and N_2_ for ESOGU-3 at 100 kPa are 3.82 (16.81%, wt), 0.697, and 0.053 mmol/g, respectively. The results show that ESOGU-3 displays selectivity toward CO_2_ over CH_4_ and N_2_ gases. The CO_2_/CH_4_ and CO_2_/N_2_ selectivities (*S*) are calculated by using single-component adsorption isotherm data. Specifically, the selectivity is determined as the ratio of the amount of CO_2_ adsorbed at 15 kPa to the amount of the other gas (CH_4_ or N_2_) adsorbed at 75 kPa.^23^ The CO_2_/CH_4_ and CO_2_/N_2_ selectivities are calculated to be 8.84 and 72.56, respectively, at 273 K and are comparable to the values reported for several other MOFs.^14,41,42^ The selective and high CO_2_ adsorption capacity of ESOGU-3 can be attributed to the presence of amide-functionalized groups within its pores. These groups enable strong quadrupolar interactions with CO_2_ molecules (quadrupole moment: 13.4 × 10^–40^ Cm^2^), thereby enhancing CO_2_ affinity and uptake. Additionally, the kinetic diameters of the gas molecules may also influence the selective adsorption. Moreover, the but-2-ene (C=C bond) group on the backbone of BDPFA^4–^ linker can contribute to the adsorption of CO_2_ through weak interactions with the electron-deficient regions of CO_2_. Additionally, despite its relatively low surface area, the CO_2_ adsorption capacity of the compound surpasses that of several well-known nonfunctionalized MOFs, a performance attributed to the presence of amide functional groups within the framework.^43,44^ Compared to previously reported anionic MOFs, ESOGU-3 exhibits a higher or comparable CO_2_ adsorption capacity. For instance, an anionic In­(III)-MOF (HBU-22) demonstrated a relatively low CO_2_ uptake of 33.39 cm^3^/g at 273 K and 1 bar, which is significantly lower than that of ESOGU-3 under the same conditions.^45^ Similarly, Pal et al. reported an anionic Co­(II)-MOF (IITKGP-6) with an interpenetrated structure, which showed a CO_2_ uptake of 50.6 cm^3^/g at 273 K and 1 bar again, lower than that of ESOGU-3.^24^ However, when compared with ESOGU-2, which shares a similar structural framework and pore architecture, ESOGU-3 exhibited reduced CO_2_ adsorption. This decrease is likely due to variations in the chemical environment of the pore surfaces. Specifically, ESOGU-2 contains polarizable sulfur atoms within its pores, which can interact more strongly with the quadrupole moment of CO_2_ molecules, thereby enhancing the adsorption relative to ESOGU-3. The CO_2_ adsorption–desorption isotherms are also measured at 293 K, and the CO_2_ isotherms recorded at 273 and 293 K are used to calculate the CO_2_ isosteric heat of adsorption (*Q*_st_) using the virial equation.^38^ The isosteric heat of CO_2_ adsorption increases as the CO_2_ loading increases, which can be due to the flexibility of pores, and the CO_2_ isosteric heat of adsorption is about 26.0 kJ/mol, which is comparable with amide-containing MOFs (Figure S3).^15,46,47^ The results show the moderate interaction between CO_2_ and ESOGU-3. The PXRD pattern of ESOGU-3 after gas adsorption indicates the structural stability of the framework and the presence of physical adsorption (Figure S4). In addition, SEM images before and after CO_2_ adsorption show that the prismatic-shaped morphology of ESOGU-3 was not changed, indicating the stability of the framework (Figure S5).

**Figure 6:**
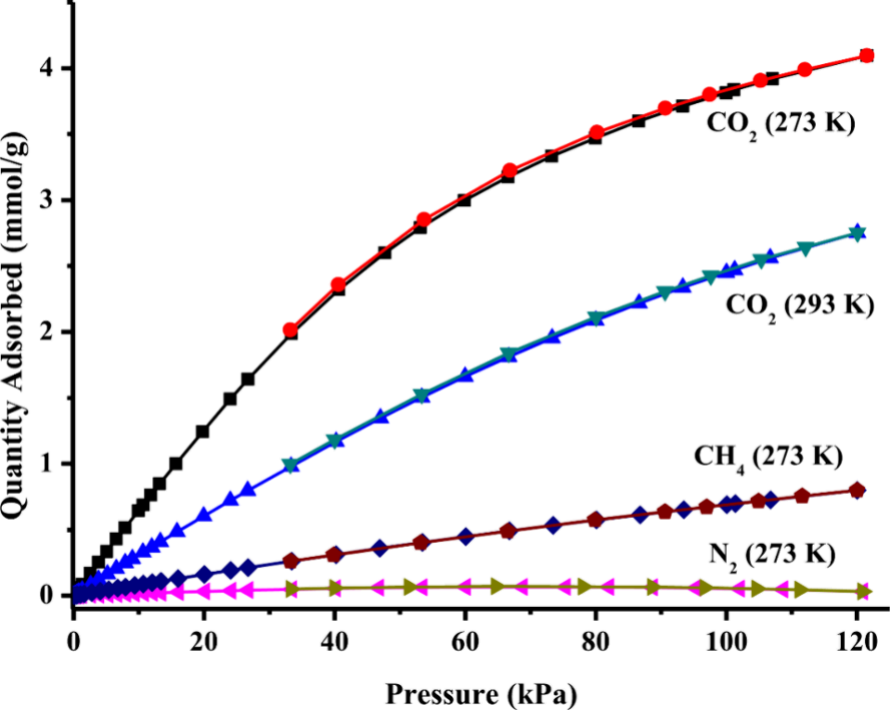
Single-component CO_2_, CH_4_, and N_2_ adsorption–desorption isotherms for ESOGU-3.

### 3.3 Dye Adsorption Results

Due to its structural stability and porosity, the dye adsorption performance of ESOGU-3 was investigated using aqueous solutions containing anionic dyes (MO and CR) and cationic (RhB and MB) dyes with varying molecular sizes. As shown in Figure 7, the highest adsorption capacities for MO, CR, MB, and RhB were found to be 4.64, 45.15, 142.16, and 29.92 mg g^–1^, respectively. ESOGU-3 demonstrated negligible adsorption of MO and CR, which was attributed to electrostatic repulsion between the negatively charged framework (zeta potential value: −35.2 mV) and the anionic dyes. In contrast, ESOGU-3 displayed effective adsorption toward the smaller MB dye via electrostatic interaction. However, RhB was not adsorbed effectively, likely due to its larger molecular size, suggesting that ESOGU-3 exhibited size-selective adsorption for cationic dyes. Owing to ESOGU-3’s highest adsorption capacity among the tested dyes, MB was selected for subsequent experiments. The color changes of the solid ESOGU-3 and the dye solution before and after MB adsorption are shown in Figure S6. Upon immersion in the MB solution, the yellow color of ESOGU-3 turned green, while the characteristic blue color of the MB solution faded.

**Figure 7:**
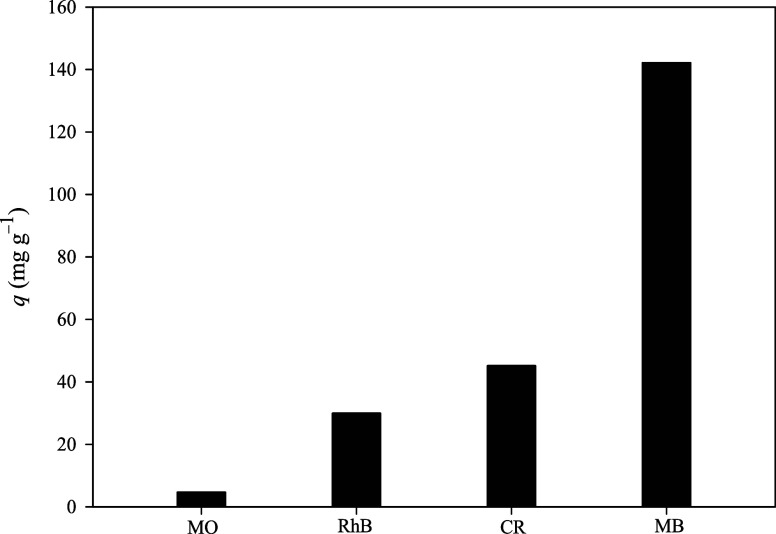
Comparison of the adsorption capacity of ESOGU-3 toward various dyes.

The pH is an important factor affecting the adsorption process, as it influences ESOGU-3’s surface charge and the chemical form of the adsorbate. To investigate the impact of pH on adsorption, the pH of the ESOGU-3 aqueous solution containing MB (100 mg L^–1^) was adjusted from 6.0 to 12.0 using different concentrations of NaOH and HCl solutions. As seen in Figure 8, the highest adsorption amount of MB was found to be 141.95 mg g^–1^ at pH = 6.0. The surface charge of ESOGU-3 in the deionized water was measured using zeta potential measurements (−35.2 mV). This result makes it possible to predict that ESOGU-3 with negative charge density will show affinity toward positively charged chemical species like MB as determined by X-ray results. Figure 8 observes that after pH 6.0, the adsorption amount of MB decreased dramatically up to pH 10.0. At lower pH values, partial degradation of the framework may occur, adversely affecting the adsorption performance. In addition, the decrease in MB adsorption at low pH values can be attributed to the formation of MBS^+^OH^–^ species on the adsorbent surface. This phenomenon is likely due to the substitution of Cl^–^ ions in the MB structure with OH^–^ ions at elevated hydroxide concentrations, which diminishes the interaction between the dye and ESOGU-3.^48^

**Figure 8:**
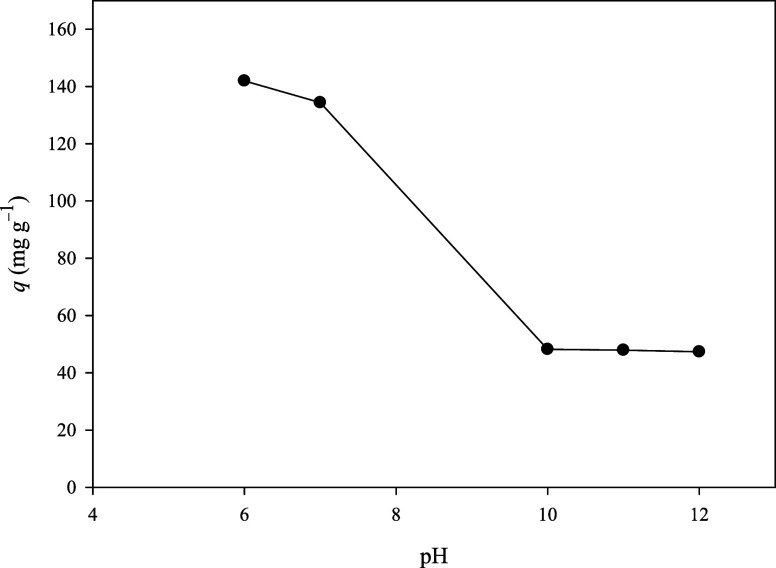
Removal of MB at various pH values.

Adsorbent dosage plays a significant role in determining the optimal conditions for adsorption. To assess the effect of adsorbent dosage on the adsorption process, ESOGU-3 dosage was varied from 0.5 to 2.0 g L^–1^ and added into 100 mg L^–1^ and 20 mL of MB dye at room temperature (Figure 9a). After 60 min of mixing, the dye concentrations in aqueous media were measured using a UV–visible spectrophotometer. The results demonstrated that the adsorption yield of MB escalated from 78.76 to 82.39% when the dosage of ESOGU-3 was augmented from 0.5 to 0.625 g L^–1^. This improvement was attributed to the increase in available surface area and the formation of additional active sites for adsorption. Beyond this dosage, the adsorption equilibrium was established and no further improvement in adsorption yield was observed. Therefore, 0.625 g L^–1^ was selected as the dosage of ESOGU-3 to be used in further experiments. The effect of temperature on the adsorption performance of MB from the aqueous solution by ESOGU-3 was also examined within a temperature range of 15–45 °C. The results, illustrated in Figure 9b, indicated that temperature variations within this range had no significant impact on the adsorption efficiency. Based on this observation, 20 °C was chosen for the next steps, as it provided strong adsorption performance without requiring extra energy, making the process both effective and energy-efficient.

**Figure 9:**
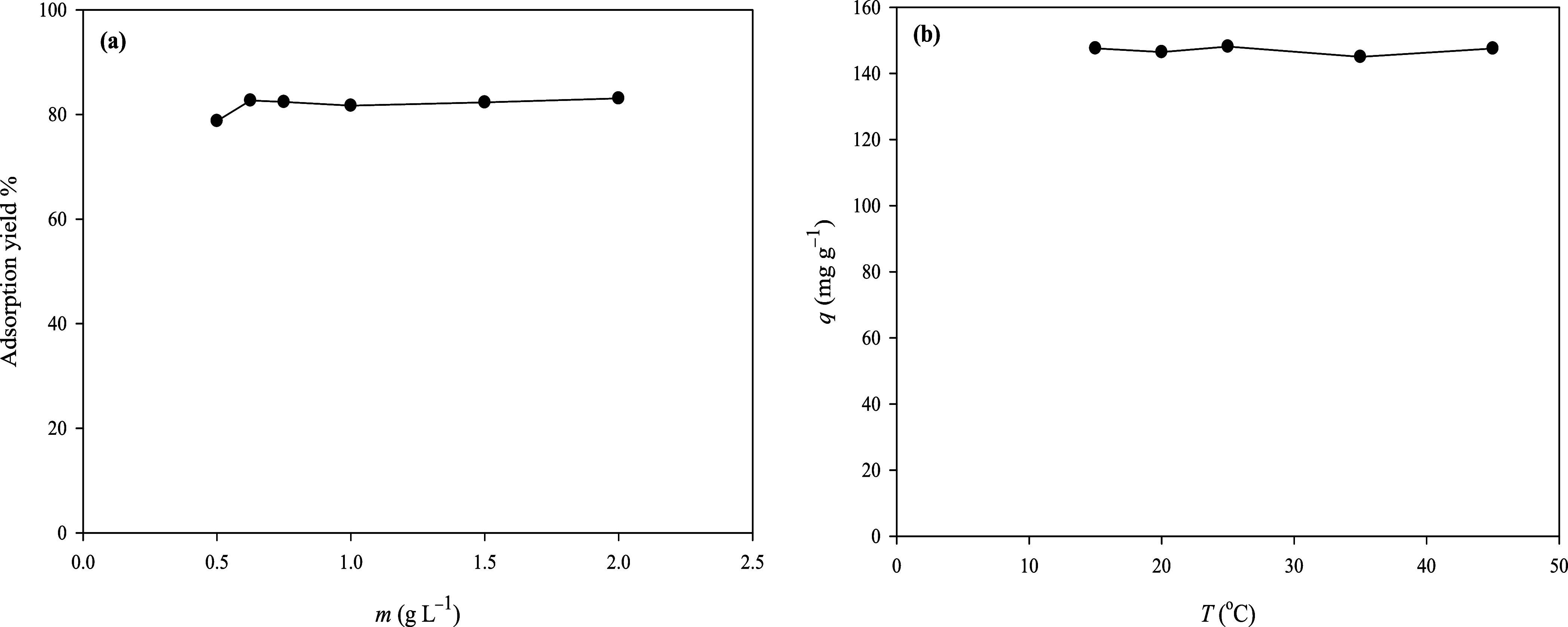
Removal of MB at various ESOGU-3 dosages (a) and temperature (b).

The effect of contact time on the adsorption process is presented in Figure S7. Adsorption experiments were conducted by adding 0.625 g L^–^^1^ of ESOGU-3 to 20 mL of MB dye solution with an initial concentration of 100 mg L^–^^1^. The mixtures were agitated for varying durations ranging from 1.0 to 90 min. As shown in Figure S7, the adsorption capacity of ESOGU-3 increased with time and reached equilibrium at 50 min. This relatively short equilibrium time indicates a rapid adsorption rate, which is an important indicator of the adsorbent efficiency. The fast attainment of equilibrium highlights one of the key advantages of the ESOGU-3 adsorption system.

The experimental data for removing MB dye from the aqueous solution using ESOGU-3 were analyzed using the Lagergren-first-order, pseudo-second-order, and intraparticle diffusion kinetic models to elucidate the adsorption mechanism. The kinetic parameters and correlation coefficients (*r* ^2^) calculated for MB adsorption are presented in Table S2. Analysis of the results revealed that the adsorption of MB ions was best described by the pseudo-second-order kinetic model. The plot representation of the pseudokinetic model is provided in Figure S8a. This finding suggests that the rate-limiting step in the adsorption process is chemisorption, involving valence forces through the sharing or exchange of electrons between the adsorbent and the adsorbate.

Adsorption isotherms are fundamental for elucidating the interactions between the adsorbate and adsorbent, thereby playing a critical role in optimizing adsorption-based treatment processes. Accurate determination of the equilibrium relationship is essential for the effective design and operation of such systems. In this study, the adsorption behavior of ESOGU-3 was evaluated using the Langmuir and Freundlich isotherm models,^49,50^ as presented in Table S3. Additionally, Table S3 summarizes the isotherm parameters and corresponding correlation coefficients for MB adsorption onto ESOGU-3. As shown in Table S3 and Figure S8b, the Langmuir model had a much higher correlation coefficient (*R* ^2^ = 0.994) compared to the Freundlich model (*R* ^2^ = 0.571), suggesting it provides a better description of how MB interacts with ESOGU-3. These point to a monolayer adsorption process, with ESOGU-3 showing a high maximum uptake capacity (415.78 mg g^–1^). Moreover, the dimensionless separation factor (*R*_*L*_) offers a straightforward way to gauge how favorable an adsorption process is. As presented in Table S3, the calculated *R*_*L*_ value for MB adsorption onto ESOGU-3 was 1.37 × 10^–2^, pointing to a highly favorable adsorption process. Table 2 presents a comparison of the *q*_max_ values of various MOFs for MB dye reported in recent years. As seen in Table 2, an anionic Cd­(II) compound with a BDPFAH_4_ linker was previously reported by our group. The MB adsorption capacity of this Cd­(II) compound (689.93 mg/g) was higher than that of ESOGU-3. In the Cd­(II) compound, the anionic framework was formed by the coordination of Cd­(II) with the BDPFA^4–^ linker and charge neutrality was maintained by dimethylamine cations located in the pores. The zeta potential value of the Cd­(II) compound (−52.6 mV) was 1.5 times higher than that of ESOGU-3, which is attributed to the presence of the complementary ion. As a result, the Cd­(II) compound displayed a higher MB adsorption than ESOGU-3. However, ESOGU-3 showed greater MB adsorption capacity than did the anionic Zn­(II)-MOF (ZJU-64-Me, 232.98 mg/g). This enhanced adsorption was likely due to its higher surface negative charge density compared to ZJU-64-Me (−24.5 mV). These results highlighted the strong potential of ESOGU-3 as an effective material for water treatment, especially in efficiently removing MB. Overall, the selective and high adsorption of MB over the anionic dye can be primarily attributed to electrostatic interactions between the negatively charged ESOGU-3 and the cationic MB. Additionally, size-selective adsorption was observed, favoring the smaller MB molecules over the larger RhB. Weak interactions such as hydrogen bonding and π···π stacking also contribute to the adsorption of MB. The PXRD pattern of ESOGU-3 after MB adsorption remained similar to that of the pristine compound, indicating the robustness of the framework (Figure S4). Moreover, SEM images of ESOGU-3 after MB adsorption showed no appreciable change in its surface morphology (Figure S5).

**Table 2:** Comparison of MB Adsorption Capacities Using Different MOFs

adsorbent	*q*_max_ (mg/g)	Reference
Ni-MOF/MXene (NM@MX)	1012.37	^51^
MOF-199/CCF	659.6	^52^
ZnCrFeO_4_/HKUST-1	32.46	^53^
UiO-66-NH_2_	549.6	^54^
ZIF-8- helicoidal electrospun polymer strips	151.6	^55^
MIL-100(Fe)@SBA-15	38	^56^
{[NH_2_(CH_3_)_2_]_2_[Cd(μ_4_-L)]·1.5H_2_O·0.75DMF}_n_	689.93	^30^
ZJU-64-Me	232.98	^36^
**ESOGU-3**	**415.78**	**this work**

## 4 Conclusions

ESOGU-3 was prepared with the amide-containing tetracarboxylic acid and a bis­(imidazole) linker. It formed a 2-fold interpenetrating 3D framework with an anionic skeleton, where the bis­(imidazole) linker contributed to charge balance through protonation of the imidazole groups. The porous ESOGU-3 displayed high CO_2_ uptake (16.81% at 1 bar and 273 K) compared to CH_4_ and N_2_, which could be assigned to Lewis acid–base interactions between the amide groups within the pores and CO_2_. Moreover, ESOGU-3 effectively adsorbed the small cationic MB dye with an adsorption capacity of 415.78 mg/g, driven by an electrostatic interaction between MB and the framework. In contrast, ESOGU-3 exhibited no affinity toward the larger cationic RhB dye, indicating size-selective adsorption. Moreover, ESOGU-3 did not adsorb either the smaller MO or the larger CR dyes due to the electrostatic repulsion between the anionic dyes and the negatively charged framework. As a result, the water-stable, porous ESOGU-3 shows promise as an adsorbent for the selective capture of CO_2_ and MB dyes.

## Supplementary Material



## Notes

Experimental details, tables for bond distances, angles and hydrogen bong geometry and adsorption, FT-IR spectra, TGA curves, SEM images, isosteric heat of CO_2_ adsorption curve (PDF)
