# Supplementary material for: An Interpenetrated Anionic MOF Featuring Amide-Functionalized Pores for CO2 and Methylene Blue Adsorption
Source: Inorg Chem. 2025 Jun 24;64(26):13540–8. doi: 10.1021/acs.inorgchem.5c02360 (PMC12239086; doi:10.1021/acs.inorgchem.5c02360)
Supplement: Supplementary file 1 [file ic5c02360_si_001.pdf]

Supporting Information

An Interpenetrated Anionic MOF Featuring Amide-Functionalized Pores for CO_2 and Methylene Blue Adsorption

Tuğba Alp Arici[a,*], Melike Şevik[b] Enes Kavak[b], Mürsel Arici[b,*]

[a]Department of Chemical Technology, Emet Vocational School, Kütahya Dumlupınar University, 43700 Kütahya, Türkiye

[b]Department of Chemistry, Faculty of Science, Eskişehir Osmangazi University, 26040 Eskişehir, Türkiye

*Corresponding authors: marici@ogu.edu.tr; tugba.alp@dpu.edu.tr

S1 The physical measurements

Elemental analyzes for carbon (C), hydrogen (H) and nitrogen (N) were performed using a Leco Truespec Micro elemental analyzer. FT-IR spectra were collected using a Bruker Tensor 27 spectrometer with KBr pellets, covering the range of 4000–400 cm^{-1}. Thermal behavior of ESOGU-3 was assessed using a Perkin Elmer instrument under dry air, applying a consistent heating rate of 10°C per minute. The zeta potential, which indicates the surface charge at the solid-liquid interface, was determined using a Malvern Zetasizer Nano ZS. The pH measurements were done with a Hanna pH meter. A Shimadzu UV-2600 spectrophotometer was utilized the record the UV-Vis spectra. Gas adsorption-desorption isotherms at 273 and 293 K temperatures were obtained with a Micromeritics TriStar II Plus analyzer. Surface morphology was examined using a Hitachi Regulus 8230. We recorded powder X-ray diffraction (PXRD) patterns using a Panalytical Empyrean diffractometer with Cu-Kα radiation, scanning from 5° to 50° in 2θ. A Bruker APEX-II diffractometer was used to gather single-crystal X-ray diffraction data with Cu-Kα radiation. The intrinsic phasing method in SHELXT-2015 via OLEX2 software was used to solve the structure and all non-hydrogen atoms was refined anisotropically for using the full-matrix least-squares method based on F^2 with SHELXL-2015[1–3] and H atoms were placed into geometrically calculated positions. The solvent molecules were highly disordered and could not be modeled. Therefore, in OLEX2, the solvent masking protocol was employed to exclude the diffraction contributions of disordered solvent molecules. According to the solvent masking protocol, there were 1008 electrons in a volume of 3796 $Å^3$ in one void per unit cell. This was coherent with the presence of 2[C_3H_7NO], 4[H_2O] per Asymmetric Unit, which accounts for 960 electrons per unit cell. One of the 2-isopropyl groups (atoms C14, C15, and C16) on the pbisoix ligand was found to be disordered in the crystal structure. These atoms were split into C14A, C15A, and C16A with occupancies of 0.5, and the disorder was modeled using SADI and SIMU restraints. Topological analysis

was carried out using the TOPOSPro program, and molecular graphics were created with Mercury program[4,5].

S2 Adsorption studies

To determine the maximum dye adsorption, a number of parameters affecting adsorption process were examined. For this purpose, stock solutions of MB, RhB, MO and CR dyes with a concentration of 1000 mg L^{-1} were prepared by dissolving 1.0 g of powdered dyes homogeneously in 1000 mL of distilled water separately. The desired dye concentrations were obtained by diluting the original stock solutions. Dye removal experiments were performed with a heater and shaker, using vials holding 20 mL of dye solutions. To explain adsorption capacities of ESOGU-3 for the designated dyes, 0.625 g L^{-1} of ESOGU-3 was added in 100 mg L^{-1}, 20 mL of dyes solutions and stirred for 60 min.

The adsorption conditions for MB dye, which achieved the highest removal, were optimized. For this purpose, the effects of the parameters pH (6.0-12.0), ESOGU-3 dosage (0.5-2.0 g L^{-1}), time (1-90 min), and dye concentration (50-600 mg L^{-1}) were investigated. After the mixtures were filtrated, the concentrations of the remnant solutions were measured at 664 nm in a UV–Vis spectrophotometer.

With the equations provided in Eqs. (1), (2), respectively, the adsorption capacity of ESOGU-3 (q_e) or the adsorption yield (%) were determined.

$$q_e = \frac{V \times (C_0 - C_e)}{m} \quad (1)$$

$$Adsorption\,(\%) = \frac{(C_0 - C_e)}{C_0} x100 \quad (2)$$

where q_e was the amount of MB adsorbed on ESOGU-3 at equilibrium (mg g^{-1}), m was the amount of ESOGU-3 (g), V was the volume of MB solution (L), C_0 was the initial MB concentration (mg L^{-1}), C_e was the equilibrium MB concentration (mg L^{-1}).

Two commonly used models were applied to assess the adsorption kinetics: the pseudo-first-order (PFO) model and the pseudo-second-order (PSO) model. To understand the adsorption mechanism of MB dye onto ESOGU-3, the compatibility of the adsorption equilibrium data with the Langmuir and Freundlich isotherm models was investigated[6,7].

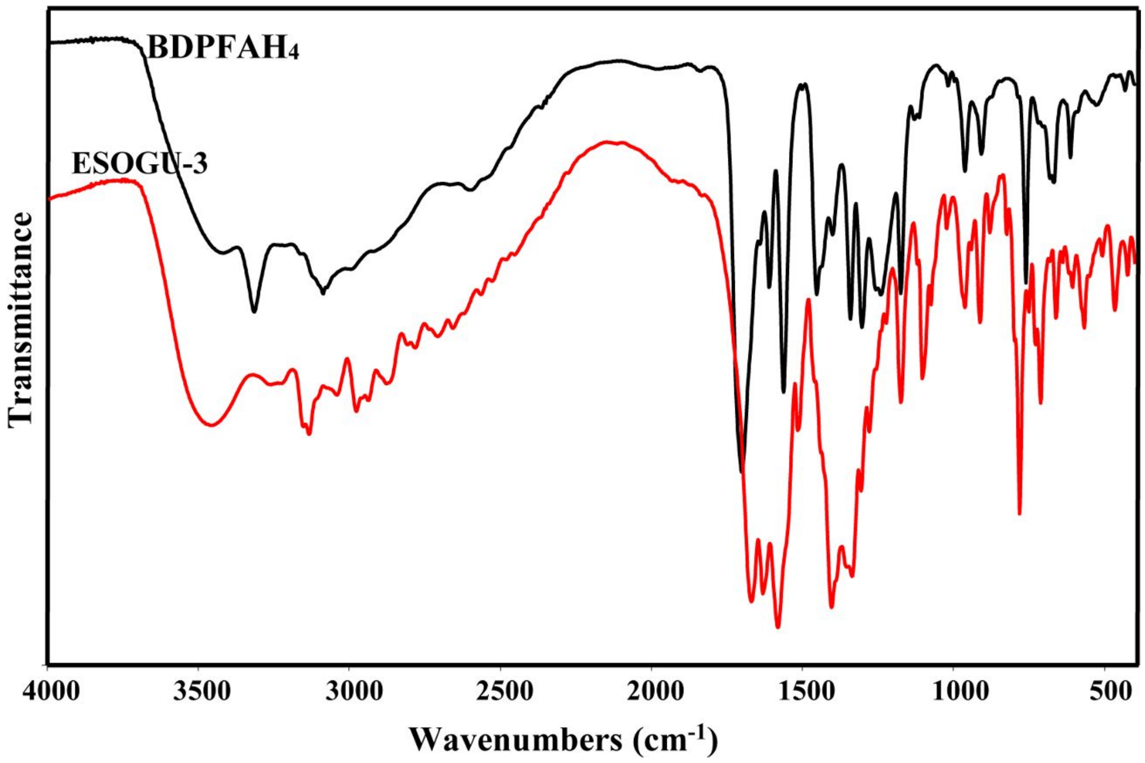

Fig. S1. FT-IR spectra of ESOGU-3 and $BDPFAH_4$ ligand

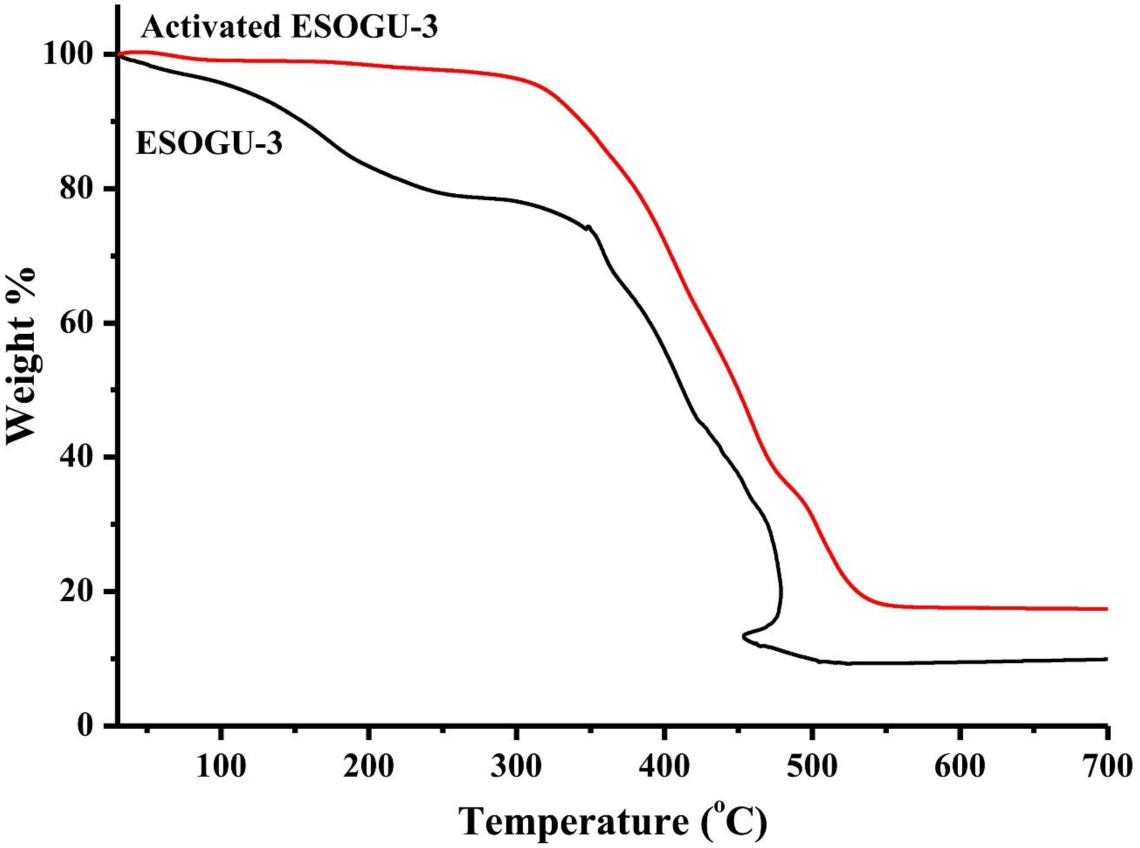

Fig. S2. TG analysis curves of ESOGU-3 and activated ESOGU-3

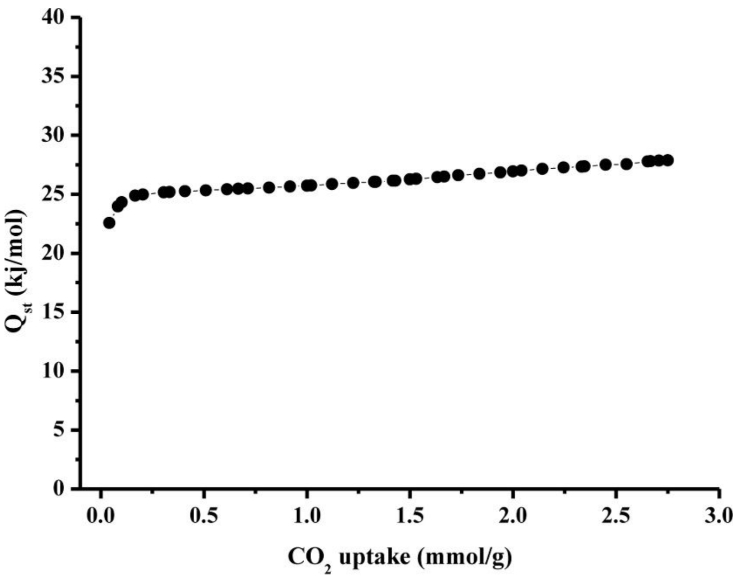

Fig. S3. Isosteric heats of CO_2 adsorption for ESOGU-3

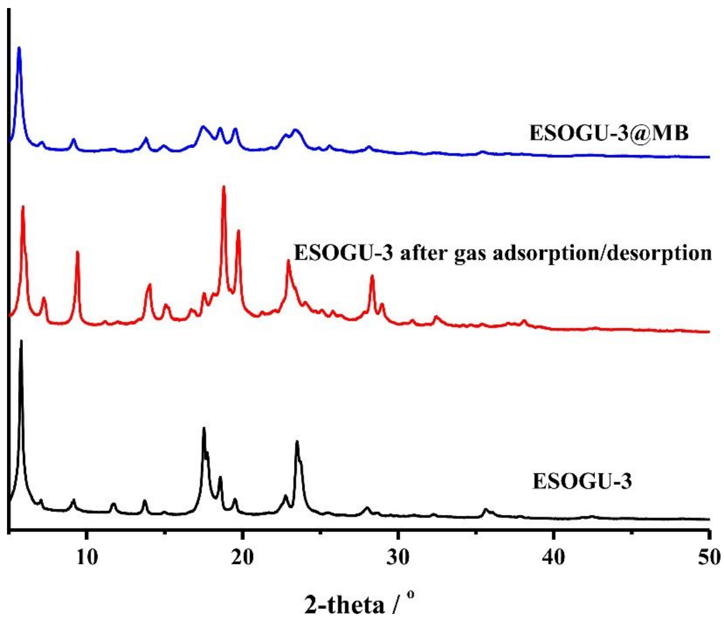

Fig. S4. PXRD patterns of ESOGU-3 after gas and MB adsorption

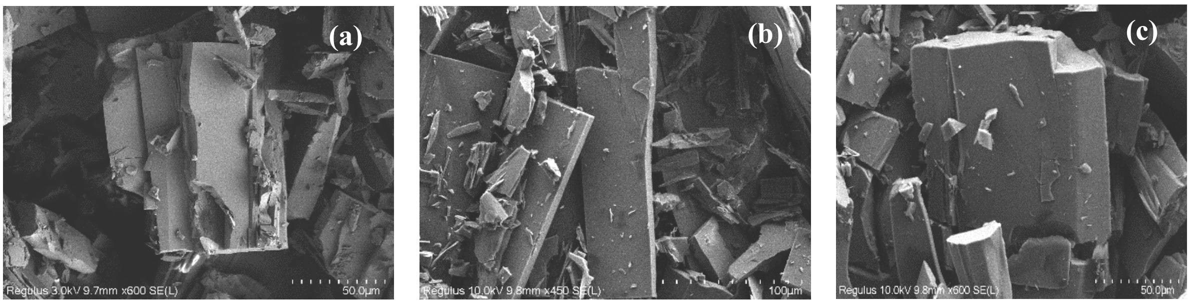

Fig. S5. SEM images of ESOGU-3 (a) after gas (b) and MB (c) adsorption.

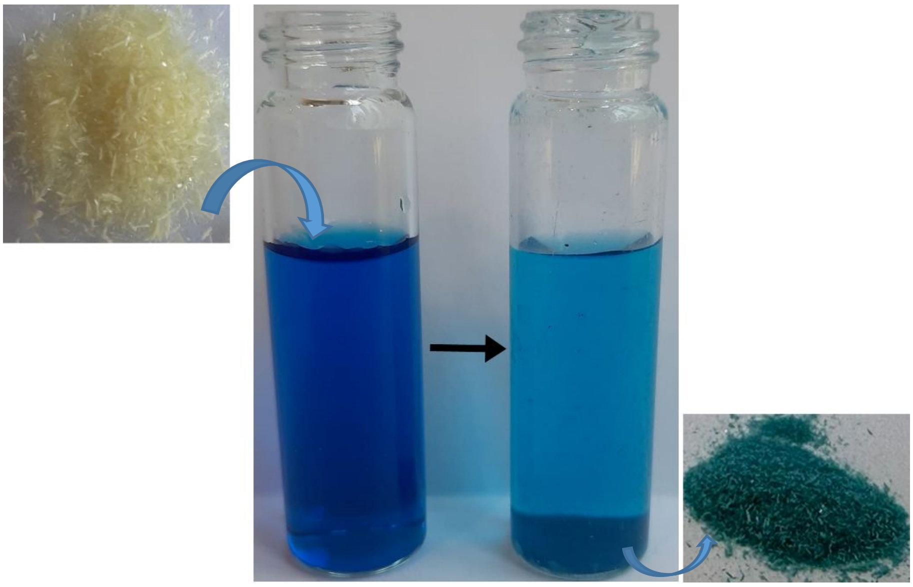

Fig. S6. The color changes of solid ESOGU-3 and MB solution before and after adsorption

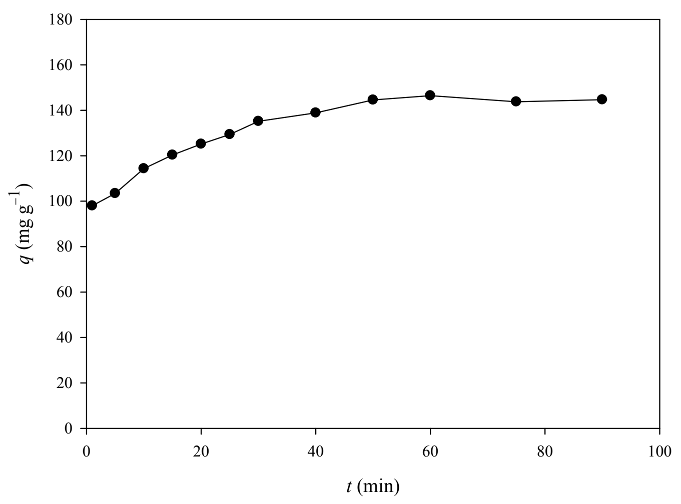

Fig. S7. The removal of MB at various contact times reveals.

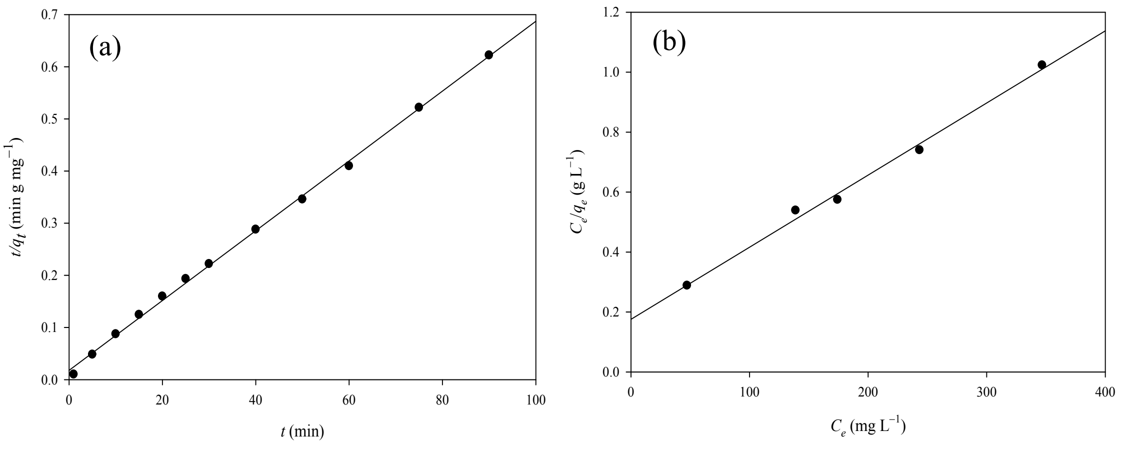

Fig. S8. Pseudo-second-order kinetic model (a) and Langmuir isotherm model (b) plots for the adsorption of MB.

Table S1. Selected bond distances, angles and hydrogen-bond geometry for the compound (Å, º)

Bond Distances				
Zn1–O4^{i}	1.9431 (16)	Zn1–O1	1.9219 (16)	
Zn1–O4^{ii}	1.9431 (16)	Zn1–O1^{iii}	1.9219 (16)	
Bond Angles				
O3^{i}–Zn1–O3^{ii}	113.77 (11)	O1^{iii}–Zn1–O3^{i}	92.75 (7)	
O1–Zn1–O3^{i}	118.65 (8)	O1–Zn1–O3^{ii}	92.75 (7)	
O1^{iii}–Zn1–O3^{ii}	118.65 (8)	O1–Zn1–O1^{iii}	122.27 (10)	
Hydrogen-bond geometry (Å, °)				
D–H···*A*	*D*–H	H···*A*	*D*···*A*	*D*–H···*A*
N1–H1···*O4*	0.86	1.81	2.668 (3)	176

Symmetry Codes: (i) *x*, −*y*+1, *z*−1/2; (ii) −*x*+1, −*y*+1, −*z*+1; (iii) −*x*+1, *y*, −*z*+1/2.

Table S2. Kinetic parameters calculated for the adsorption of MB.

PFO			PSO		
k_1 (min^{-1})	q_1 ($mg\ g^{-1}$)	R^2	k_2 ($g\ mg^{-1}\ min^{-1}$)	q_1 ($mg\ g^{-1}$)	R^2
4.04×10^{-2}	43.56	0.892	2.50×10^{-3}	149.39	0.998

Table S3. Isotherm parameters calculated for the adsorption of MB.

Isotherm model	Parameters	r^2
Langmuir	q_{max}=415.78, K_L=0.422<br>R_L=1.37×10^{-2}	0.994
Freundlich	n=2.40, K_F=66.49	0.571

S. References

(1) Sheldrick, G. M. SHELXT-Integrated Space-Group and Crystal-Structure Determination. *Acta Crystallogr. Sect. A Found. Adv.* **2015**, *71* (1), 3–8.

(2) Sheldrick, G. M. Crystal Structure Refinement with SHELXL. *Acta Crystallogr. Sect. C Struct. Chem.* **2015**, *71* (1), 3–8.

(3) Dolomanov, O. V; Bourhis, L. J.; Gildea, R. J.; Howard, J. A. K.; Puschmann, H. OLEX2: A Complete Structure Solution, Refinement and Analysis Program. *J. Appl. Crystallogr.* **2009**, *42* (2), 339–341.

(4) Blatov, V. A.; Shevchenko, A. P.; Proserpio, D. M. Applied Topological Analysis of Crystal Structures with the Program Package ToposPro. *Cryst. Growth Des.* **2014**, *14* (7), 3576–3586.

(5) Macrae, C. F.; Bruno, I. J.; Chisholm, J. A.; Edgington, P. R.; McCabe, P.; Pidcock, E.; Rodriguez-Monge, L.; Taylor, R.; Streek, J. V. D.; Wood, P. A. Mercury CSD 2.0–New Features for the Visualization and Investigation of Crystal Structures. *J. Appl. Crystallogr.* **2008**, *41* (2), 466–470.

(6) Langmuir, I. The Adsorption of Gases on Plane Surfaces of Glass, Mica and Platinum. *J. Am. Chem. Soc.* **1918**, *40* (9), 1361–1403.

(7) Freundlich, H. Über Die Adsorption in Lösungen. *Zeitschrift für Phys. Chemie* **1907**, *57* (1), 385–470.
